# Neuroscience Concepts Changed Teachers’ Views of Pedagogy and Students

**DOI:** 10.3389/fpsyg.2021.685856

**Published:** 2021-08-11

**Authors:** Zhengsi Chang, Marc S. Schwartz, Vicki Hinesley, Janet M. Dubinsky

**Affiliations:** ^1^School of Behavioral and Brain Sciences, The University of Texas at Dallas, Richardson, TX, United States; ^2^College of Education, The University of Texas at Arlington, Arlington, TX, United States; ^3^Department of Neuroscience, University of Minnesota, Minneapolis, MN, United States

**Keywords:** neuroeducation, Mind Brain and Education, professional development, educational neuroscience, teacher practice, pedagogy

## Abstract

Advances in neuroscience reveal how individual brains change as learning occurs. Translating this neuroscience into practice has largely been unidirectional, from researchers to teachers. However, how teachers view and incorporate neuroscience ideas in their classroom practices remains relatively unexplored. Previously fourteen non-science teachers participated in a 3-week three credit graduate course focusing on foundational ideas in neuroscience. The current work was undertaken to gain insight into if and how individual teachers choose to later apply the proposed set of educational neuroscience concepts (ENCs) in their classrooms. This qualitative follow-up study examined commonalities in how teachers of diverse ages and subjects utilized their new neuroscience understandings. To this end, a year after the course, all participants assessed their perceived usefulness of the ENCs in a survey. Six of those teachers permitted classroom observations and participated in interviews that focused on how the ENCs may have influenced their lesson planning and teaching. The survey revealed that irrespective of subject areas or grade levels taught, teachers found the ENCs useful as organizing principles for their pedagogy now and in the future. Overall teachers estimated that the ENCs’ influence on lesson design had increased from 51% prior to the course to an estimated 90% for future lessons. A cross-case analysis of classroom observations and interviews revealed how teachers used ENCs to inform their pedagogical decisions, organize actions in their classroom, influence their understanding of students, and respond to individual contexts. Teachers recognized the importance of student agency for engaging them in the learning process. The ENCs also offered teachers explanations that affirmed known practices or helped justify exploring untried techniques. The foundational neuroscience concepts offered a small group of teachers a lens to reconsider, re-envision and re-design their lessons. Some teachers applied these ideas more broadly or frequently than others. This case study provided insights into how teachers can directly apply neuroscience knowledge to their practice and views of students.

## Introduction

Neuroscience and education have been struggling to determine their conceptual and practical relationship for a generation (Cruickshank, 1981; Bruer, 1997; Goswami, 2006; Meltzoff et al., 2009; Carew and Magsamen, 2010; McCandliss, 2010; Sigman et al., 2014; Bowers, 2016; Howard-Jones et al., 2016; Feiler and Stabio, 2018). Some researchers (Bruer, 1997; Bowers, 2016) argued that bridging neuroscience and education requires training in science, thus the translation of neuroscience to education cannot be implemented by teachers, but via middle ground domains such as psychology. Neuroscientists investigate the neurophysiological underpinnings of behaviors that are fundamental for education like learning, memory, attention, motivation, etc. and of disorders relevant to education like ADHD, dyslexia, dysgraphia, etc. (Goswami, 2006; Sigman et al., 2014; Howard-Jones et al., 2016). Cognitive scientists investigate knowledge construction from theoretical, neurophysiological, and behavioral perspectives (Howard-Jones et al., 2016). Educational psychologists and researchers investigate the knowledge, pedagogy, and best classroom practices needed by teachers (Good and Brophy, 1995; Im et al., 2018; Lavigne and Good, 2019). Therefore, in the debate over how neuroscience should influence education, teachers often take a secondary role. While teachers’ desires to learn neuroscience have been documented and acknowledged (Pickering and Howard-Jones, 2007; Hook and Farah, 2013), teachers participate as subjects in the research endeavors or do small scale action-research on their own (Churches et al., 2020; Wright, 2020) but are rarely granted agency in conducting research (Juuti et al., 2021) or in determining how neuroscience could, should, or does influence education (Dubinsky et al., 2013; Tan and Amiel, 2019).

The *researcher-initiated* neuroscience applications have also generated philosophical and pragmatic issues for teachers. The first general epistemological concern is that laboratory findings do not easily translate into classroom recommendations because classroom dynamics are too complex and fluid to assume that researchers can offer teaching decisions to teachers (Bruer, 2006; Bowers, 2016). A second related pragmatic issue involves the manner in which teachers acquire neuroscience knowledge, through a didactic process or a constructivist one. Within a didactic setting, teachers are more likely to need translation and guidance to understand the neuroscience (Tham et al., 2019) or to be told how neuroscience should be translated into practice (Hardiman, 2012; Churches et al., 2017). Within a constructivist setting, teachers may make personal meaning by combining the neuroscience and their own insights to the relevant contexts of their classrooms (Pickering and Howard-Jones, 2007; Dubinsky, 2010; Dommett et al., 2011; Hardiman et al., 2012; Dubinsky et al., 2013, 2019; Hook and Farah, 2013; Tan and Amiel, 2019).

We recognize the *teacher-initiated* application as a promising approach since teachers ultimately make all classroom decisions (Bishop, 2008). In making classroom decisions, teachers consider their knowledge of students, theoretical understanding of education and beliefs as well as immediate and planned classroom goals and actions (Shavelson and Stern, 1981; Clark and Peterson, 1986; Shulman, 1987; Schoenfeld and Kilpatrick, 2008). Teacher beliefs may act as filters for interpreting events, frames for conceptualizing a teaching strategy or problem, or guides for intended or immediate actions (Fives and Buehl, 2012). Despite the contemporary emphasis promoting rational, data-based decision making (van der Scheer et al., 2017), many teachers rely heavily upon intuitive expertise, gleaned from years of experience, to make classroom decisions (Vanlommel et al., 2017). Indeed, teachers’ own theories regarding teaching and learning often drive their decision making (Bishop, 2008; Borko et al., 2008; Bullock, 2011). While teachers can be successfully trained to use and evaluate student data as a basis for decision making (van der Scheer et al., 2017), such data is not always available for consultation when decisions have to be made in the moment during instructional interactions. Teachers learn from the trial and error process of their own teaching year after year. Evaluating such personal experiences and classroom data within professional learning communities can produce insights supporting evidence-based decision making, a process termed action-research (Little, 2007; Wright, 2020). These endeavors emphasize teacher agency in applying their broad background knowledge of students, best practices, and current policies to instructional practices.

The current study pursues this *teacher-initiated* perspective, where teachers determine if or how neuroscience influences their situated pedagogical decisions. Clement and Lovat (2012) asserted that only teachers could truly demonstrate whether neuroscience could influence education. In their view, the ultimate test for the relevance of neuroscience to education should be whether neuroscience knowledge provides teachers with “usable knowledge” that can affect the pedagogical decisions they make in their own classroom practices. Neuroscience may not provide immediately useful knowledge for classroom teaching, because neuroscience describes natural phenomena and processes while education prescribes pedagogical decisions to improve learning outcomes. However, neuroscience may indirectly affect education by bringing insights into teaching and learning that take into account the biological and physiological constraints upon these processes imposed by our brain and body. Such background information falls under Shulman’s designation of *Knowledge of Students* as one of the seven necessary categories of teacher knowledge (Shulman, 1987). Clement and Lovat (2012) further argued that once neuroscience knowledge is shared in a more accessible way, teachers could play a critical role in identifying what neuroscience knowledge is pertinent and applicable to their own classroom decisions and practices.

A variety of outcomes have been reported from programs which introduce neuroscience knowledge to teacher audiences. At the knowledge transfer level, teachers want the neuroscience explained in an accessible and easily applied manner (Tham et al., 2019). A short didactic neuroscience introduction may cause teachers to think about the teaching habits they had acquired (Howard-Jones et al., 2020). Formal teacher preparation programs have embraced inclusion of neuroscience as fundamental background knowledge (Deans for Impact, 2015) and are beginning to evaluate those enactments (Friedman et al., 2019; Luzzato and Rusu, 2019). For in-service science teachers, professional development (PD) programs in neuroscience influence pedagogy, in that classroom observations revealed increased inquiry-based pedagogy and improvements in the classroom cognitive environment characterized by increased higher-order thinking, deep knowledge, substantive conversations, and connections to real world problems (MacNabb et al., 2006; Roehrig et al., 2012). In-service neuroscience PD resulted in increases in self-reported teacher self-efficacy and increased use of student-centered practices (Brick et al., 2021a). However, the PD in the latter three studies was also delivered utilizing constructivist approaches, so the teachers’ behavioral and pedagogical changes may not be attributable to neuroscience alone. These studies do suggest that neuroscience knowledge might be influential in convincing teachers of the merits of constructivist teaching approaches. In focus and lesson study groups, teachers connect ideas from basic neuroscience to their own pedagogical practices (Dubinsky et al., 2013; Tan and Amiel, 2019; Tan et al., 2019). After a master’s course focusing upon the neuroscience of learning and memory, non-science teachers explained their revisions to a lesson plan utilizing neuroscience ideas (Schwartz et al., 2019). Understanding biologically how stress and trauma can suppress learning, teachers self-reported curtailing harsh disciplinary practices and providing more student social and emotional support (Brick et al., 2021b). In the reflective and iterative lesson study process, providing grade school teachers with understanding of neuroscience principles guided them to shift their pedagogies to more student-centered practices and afforded them with the means to explain those choices (Tan and Amiel, 2019; Tan et al., 2019). These studies suggest that neuroscience ideas may indeed have influenced teachers’ pedagogical choices. However, the majority of measures reported to date were either strictly observational, planned, or self-reported, after-the-fact information about pedagogy. Only the lesson study research, which included observation, mentored feedback and reflection (Tan and Amiel, 2019; Tan et al., 2019), has addressed if or how the neuroscience ideas influenced teachers’ thinking and actions regarding their instructional choices as they were teaching.

To explore if and how teachers translate into practice neuroscience ideas encountered in coursework, observation of their classroom implementations combined with their explanations of those applications are required. Such teacher-initiated actions would constitute evidence to satisfy the criteria established by Clement and Lovat (2012) for a direct connection between neuroscience and education. To do this, we revisited a cohort of teachers who had taken a three credit graduate course taught using a constructivist approach to develop teacher basic neuroscience knowledge (for details of the intervention, see Schwartz et al., 2019).

In the course evaluation, we used ten Educational Neuroconcepts (ENCs) crafted for an audience of educators in areas such as memory, learning and emotions. These neuroconcepts represent a synthesis of neuroscience research that offers insight into basic or general principles of how the brain creates behaviors (Supplementary Table 1; Society for Neurosicence, 2008; Dubinsky et al., 2013). The ENCs are more than a series of independent concepts teachers need to understand about how brains function. Together they represent the complexity of human brains and neuroscience function. No one concept captures the entirety of brain function, nor do all ten. The ENCs were written as an overview of the foundational neuroscience knowledge for teachers to understand how learning occurs, and how memory, emotions and context mediate learning in their students’ brains. In their initial conception, teachers were free to navigate the ENCs in the context of their own practices as their understanding of neuroscience permits (Dubinsky et al., 2013). How these principles might operate to influence teacher thinking and execution of their lessons remains unexplored. Were these ideas useful and applicable on a daily basis? If so, the ENCs might be appropriate for guiding content choices in pre-service neuroscience coursework or in-service PD. If not, then the usefulness of spending precious training time on neuroscience would be questioned.

Previously, we documented how the ENCs influenced teachers’ thoughts about their lesson planning (Schwartz et al., 2019). The course did not claim that neuroscience justified any particular classroom approach. Applications of neuroscience ideas were left to the teachers who have expertise in the art of teaching and how to consider the contexts and policies of their specific classrooms. While the study demonstrated that for 14 mostly non-science teachers, the ENCs had a powerful impact on their thinking about the nature of effective pedagogy, we did not know whether some, all or any of the ENCs would have lasting power in influencing what teachers actually did in their classroom or the extent to which the ENCs were used to explain or rationalize classroom decisions. More formally, the current research question addressed how the ENCs influenced teachers’ thinking and practices over time. To this end, a year after the course, we surveyed the same 14 teachers to assess their perceptions of the usefulness of the ENCs. Six of those teachers permitted classroom observations and participated in interviews that focused on how the ENCs influenced their teaching and classroom decisions.

We hypothesized that the ENCs would have an enduring role in influencing teachers’ thinking or classroom decisions regarding their pedagogy, and that the ENCs would not dictate teacher pedagogy in any specific way. However, as in all qualitative work, we recognized that competing or rival hypotheses might also explain changes observed in teacher behavior or thinking (Yin, 2003). We explore these alternative hypotheses in the discussion section.

## Materials and Methods

Evaluating the long term impact of PD is a challenging endeavor, often relying on surveys, interviews and teacher self-reports (Garet et al., 2001; Penuel et al., 2007; Colbert et al., 2008; Ravhuhali et al., 2015). While observations are more challenging to organize and conduct, they also play a valuable role in evaluating PD (Guskey, 2002; King, 2014; Stecher et al., 2018; Tan and Amiel, 2019). Here we combine the advantages of surveys to provide the teachers’ understanding of the perceived importance of ENCs in pedagogy, and classroom observations and interviews to describe the influence of the ENCs in teachers’ pedagogical decision-making. We used a cross-case analysis of these teachers’ interpretations and application of the ENCs to reveal if or how the ENCs influenced the teachers’ practices.

### Context

The study took place as a follow-up to a master’s level course, Neuroscience for Educators, offered as an elective in a Mind Brain and Education program at a Midwestern university in May 2016. Details of the course were presented previously (Schwartz et al., 2019). Given that the teachers taught a variety of subjects across the entire K-12 spectrum, the course delivered content on the neurobiological basis of learning and memory using lessons appropriate for K-12 classrooms (MacNabb et al., 2006). Inquiry and active learning pedagogies were employed to model best teaching and PD practices (MacNabb et al., 2000; Garet et al., 2001; Desimone, 2011; Dubinsky et al., 2013; Darling-Hammond et al., 2017). Comparable neuroscience content and delivery have been evaluated as part of in-service science teacher PD (MacNabb et al., 2006; Roehrig et al., 2012; Dubinsky et al., 2013, 2019; Schwartz et al., 2019; Brick et al.,2021a,b). Briefly, the topics covered included general brain structures and their functions; neurons, synapses, and circuits; synaptic plasticity; autonomic nervous system and emotions; homeostasis; memory, learning and effects of drugs on brain function; social and emotional learning; epigenetics of learning and memory and nature vs. nurture; brain development; reading and circuit formation; ADHD and dyslexia. Approximately 20% of the course was lecture-based with the remainder utilizing active learning strategies such as questioning; discussions; modeling; dissections; short, independent station activities; and group guided and open inquiry that included data gathering, analysis, interpretation and communication. After each lesson, participants discussed the pedagogy demonstrated, how they learned the material, and how they might employ comparable pedagogy in their own practices. Participants were encouraged to make connections between the neuroscience and their teaching practices but such connections were not provided by instructors. Daily reflections captured what the neuroscience content meant to each participant. Lessons plans used in the course and demonstration videos from comparable PDs are available online (MacNabb et al., 2006). During the course, the ENCs (Supplementary Table 1) were not taught didactically but were used in assessments, so teachers did see them as a list of neuroscience concepts (Schwartz et al., 2019).

### Participants

The study was formally approved by the Institutional Review Board of the University of Texas at Arlington. All 14 teachers who participated in the course voluntarily and formally consented to participate in a follow-up survey, 1 year after the course. All participants provided their written informed consent to participate in this study. Six teachers (Table 1) voluntarily consented in writing to be observed in their classrooms and interviewed afterward. Written consent or oral approval was obtained from school heads or administrators as dictated by district policies. Sample size was saturated, being limited by the initial course enrollment and the consenting process.

**TABLE 1:** Teacher profiles.

Pseudonym	ENC used	Subjects Taught	Grade	Bilingual	FRL
Ms. Able	1, 4, 5, 8, 10	Language Arts	Pre-Kindergarten	No	69%
Mr. Ruiz	1, 2, 4, 5, 10	Math and Science	5	Yes	69%
Ms. Gomez	1, 2, 6, 9	Math	1	Yes	58%
Ms. Bell	1, 2, 3, 4, 5	Math	High school	No	1%
Ms. Crow	7, 8, 9, 10	Language Arts	4	No	69%
Ms. Lake	4, 5, 8, 10	Reading-Language Arts	K-5	No	39%

*ENC used refers to ENCs the teachers noted in their pre-observation forms.*

*Bilingual, whether the teacher can provide instruction in both English and Spanish.*

*FRL, percentage of school population eligible for free or reduced price meals. Numbers were taken from school district websites. All other information was provided by the teachers.*

### Data Collection

The data collection occurred late in the spring semester of 2017, toward the end of the academic year, approximately 11 to 12 months after the course. A mixed methods approach was employed to provide multiple data sources for gaining insights and forming conclusions. Thus, a survey, classroom observations and interviews have been utilized.

### Survey

The survey probed the extent to which a teacher applied each of the ENCs in their practice on a scale of 0 to 100 (see Supplementary Material). For each ENC, teachers were asked to assess the degree of application in their pedagogy prior to taking the course, currently and in the future. By assessing perceptions at a single time point, the relative importance of the ENCs at present, past and future times can be more accurately judged and compared (Howard, 1980; Levinson et al., 1990). Any usefulness scale might drift over the course of a year if the survey had been administered in a pre-post design. With teachers’ current knowledge of the ENCs and a year of using them, views regarding the ENCs would be expected to reflect their experiences both in and after the course. A response of 0 triggered the additional question: “If you are unlikely to apply this concept, what are the obstacles preventing its application?” The survey was administered digitally via Qualtrics. Aggregate survey data were returned to participants attending a reunion held at the end of the school year, where they verbally confirmed the findings.

### Observations

For six teachers, a classroom observation was conducted to demonstrate how the influence of ENCs played out in real classroom contexts. Prior to the observation, each teacher filled out a two-question form, describing the lesson to be viewed and its place within the unit, and listing which ENC(s) influenced the lesson and why. The pre-observation forms were submitted via email before the classroom observations took place. The classroom observations were conducted at a time scheduled by the teacher. Many districts were worried that observations would disrupt classes and student learning, so only one visit was made per teacher. During the observation, the observer (VH), a former teacher, noted the sequential teacher dialog and actions. Teacher, and not student, behaviors were the focus of the observations. Copies of classroom artifacts were collected as needed to understand the observed lesson. The same observer carried out all classroom visits and interviews. Field notes were written (VH) for each teacher summarizing the classroom observation and the teacher’s interview responses.

### Interviews

Post-observation interviews were conducted with the six teachers to provide insight into how they thought the ENCs influenced their teaching practice. These took place outside of class at a time convenient to the teacher and observer, either immediately in person or within 24 h over the phone, following the observations. These structured interviews lasted 30 to 90 min. During interviews, teachers answered the same set of questions regarding each neuroconcept they identified (see Supplementary Material). In answering this set of questions, teachers identified actions they took during the observation and provided explanations for how they connected the ENC being discussed to those actions.

### Quantitative/Statistical Analysis

The quantitative analysis was conducted on the survey data of all 14 teachers regarding the teachers’ use of the 10 ENCs across the three time points. A two-way repeated measures ANOVA was conducted on the rating scores as the dependent variable with “time point” and ENCs as within-subject independent variables, using IBM SPSS^®^ Statistics Version 26. Data from two teachers were discarded due to missing values. For all data analyses, the significance level was set at two-tailed *p* < 0.05. Pairwise comparisons using a Bonferroni correction were conducted as follow-up tests after significant effects were observed.

### Cross Case Synthesis/Qualitative Analysis

To better understand how teaching decisions were influenced by the ENCs, we adopted the cross-case analysis approach (Stake, 1995; Baxter and Jack, 2008; Yin, 2017). Cross-case analysis permits finding commonalities across multiple teachers in different contexts that can contribute to generalizations about how relevant neuroscience knowledge can be applied to classroom practice (Miles and Huberman, 1994; Khan and VanWynsberghe, 2008). Furthermore, this kind of analysis can help “estimate the effect” of an intervention, such as the influence of ENCs on a teacher’s pedagogy (Goertz and Mahoney, 2012, p.89). The coding and data analysis process followed a grounded theory approach (Creswell, 2013).

One author (VH) transcribed all the recorded interviews into written documents for access purposes and initially wrote individual summaries of each observation. Two authors (VH, JMD) reviewed and revised the summaries of individual cases. The summaries focused upon three components: (i) each ENC mentioned by the teacher, (ii) examples of teacher actions from the observations, and (iii) explanations of intentions provided from the interviews or pre-observation form. The purpose of this first pass analysis was to extract examples where specific ENCs were applied. A list of preliminary codes was formed from the research questions and the individual case summaries (ZC, JMD).

Following the formation of the preliminary codes, two authors (VH, MS) wrote 21 vignettes from the pre-observation forms, classroom observations, and post-observation interviews, illustrating instances in which the teachers indicated an ENC had some influence or application. The vignettes were discussed by all four authors and analyzed to refine the set of codes. Codes were sought that transcended the content of individual ENCs to address the application of ideas represented by one or more ENCs. Codes for specific pedagogical practices, e.g., working in groups, were considered lesson specific and were not likely to be universally encountered across single visits to each classroom. Evidence from interviews, observations and field notes was triangulated to identify common ways that the teachers applied ENCs, testing the set of codes. Codes were further refined, discussed, and tested iteratively by all authors until complete agreement was reached. Codes were initially considered saturated when all additional codes could be viewed as subsets of the existing set. Field notes were reviewed at this point as a check for completeness. Eight final codes emerged. Using the final list of codes, each author separately coded all the pre-observation forms, classroom observations, and the post-observation interview documents for each teacher on the ENCs they specified. Full agreement was initially achieved on 77% of the coding. Where disagreements occurred, examples were discussed, field notes were consulted and recoding continued until 100% agreement was reached. In addition, authors challenged each other to identify inconsistencies in the narratives, to find evidence that did not support the emerging themes, and to view teachers’ use of ENCs from both explicit and implicit points of view. Notes were kept on each coding discussion and writing sessions.

Once coding was complete, we returned the qualitatively derived themes to the teachers and provided them with the opportunity to agree or disagree with their transcripts, the themes, and which themes their data supported. We invited teachers to engage with us further for follow-up interviews to gather additional feedback and data and waited a month for replies. We planned to use such conversations to help us find out what we might have misinterpreted or left out. All 5 responders agreed with the researchers’ analysis but declined further interviews. The sixth teacher did not respond. While the reasons for this decline were not probed or volunteered, the added stresses of teaching during a pandemic may have contributed. Under these difficult circumstances, ethically, we did not feel that the time demands of our research agenda took precedence over teachers’ main concern for student learning. Thus, without additional teacher cooperation, the synthesized member checking process outlined by Birt et al. (2016) was executed to the extent possible.

Final code saturation (Bowen, 2008; Sim et al., 2018) was confirmed when (1) all interview and observation data had been coded, (2) researchers repeatedly encountered the same insights from different participants across data sources, and (3) the coding results were confirmed by member checks (Creswell and Miller, 2000; Bowen, 2008), with no teachers adding new insights.

The codes were subsequently grouped into three themes explored in the next section. Theoretical saturation (Bowen, 2008) was reached when additional analysis could not reveal new themes. In writing the manuscript and choosing examples to illustrate the codes, the vignettes, observations, interviews and field notes were consulted. Quotes were taken from the interviews.

A number of procedures contributed to the validity of this process (Creswell and Miller, 2000). The teachers offered rich, detailed information regarding their own thoughts in how the ENCs influenced their practices which are conveyed below. From the perspective of the researchers, the extensive discussion and triangulation described above also examined the qualitative sources for disconfirming evidence. At various points in time, descriptions of the coding process and their justifications were written to provide an audit trail and the basis for this methods section. This constituted a form of journaling. Two authors (VH, ZC) were part of the original class (but were not observed), guaranteeing the teacher lens was represented collaboratively in the analysis and writing process. The views of a knowledgeable educational researcher (GR) were sought and manuscript drafts underwent two rounds of independent peer debriefing to assure that the identified codes were supported by the data.

A number of techniques were used to ensure the trustworthiness of this cross-case analysis (Lincoln and Guba, 1985; Erlandson et al., 1993). Relationships between investigators and teachers were prolonged, built over multiple years in the MBE program, the Neuroscience for Educators course, the survey and classroom follow-up, a reunion, and the ensuing member checking. Sample size was exhaustive being limited by course enrollment and administrative permission for observations. The purposeful investigation into classroom applications of the ENCs persisted for the maximum time permitted by administrations, providing observation and interview data for triangulation. Referential materials were collected when appropriate to understanding the observed classes. Member checking was employed to the extent teachers’ remained engaged. The accuracy of the qualitative analysis was validated by peer debriefing. Internal documentation provided audit trails for the narrative descriptions of classroom events used in the qualitative analysis.

## Results

### Survey

**FIGURE 1:**
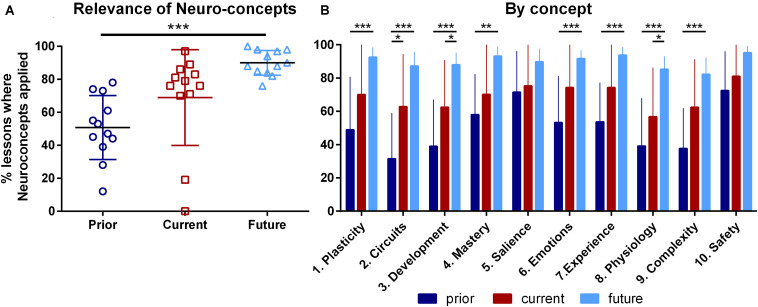
Teachers ratings of the direct applicability of 10 neuroconcepts to their teaching prior to the Neuroscience for Educators course, in the current academic year and in future lessons. **(A)** Brackets represent mean ± SD for each time point. Individual data points represent different teachers, mean ± SD summed across all ENCs. The two lowest current responses were from teachers holding administrative positions, representing their limited exposure to students. Significance shown represents Bonferroni post-tests following the significant main effect of time (*p* < 0.001, Cohen’s *d* = 16.1). **(B)** Bars represent mean ± SD for each individual ENC, summed across all teachers. Significance shown represents Bonferroni post-tests following individual one-way ANOVAs for each ENC (*p*-values < 0.001, 0.000, 0.000, 0.002, 0.14, 0.000, 0.001, 0.000, 0.000, and 0.054, respectively, for ENCs 1 to 10); *, **, *** represent *p* < 0.05, 0.01, 0.001, respectively; *N* = 12 teachers.

The survey probed the degree to which each ENC was applied in teachers’ lesson planning, currently, retrospectively prior to taking the course and prospectively in the future (Figure 1). Mauchly’s test indicated that the assumption of sphericity had not been violated for time [W (2) = 0.68, *p* = 0.15], or for ENC [W (44) = 0.001, *p* = 0.17]. Significant main effects were found for time [two-way ANOVA, *F* (2,22) = 12.26, MSE = 3798.64, *p* < .001, partial *η^2^* = 0.53], for ENC [*F* (9,99) = 7.18, MSE = 333.01, *p* < 0.001, partial *η^2^* = 0.40] and for their interaction [*F* (18,198) = 3.78, MSE = 116.84, *p* < 0.001, partial *η^2^* = 0.26]. *Post-hoc* tests following the main effect of time indicated that there was an increase in the overall likelihood of applying the ENCs in teaching practice across the three time points, averaging over all 10 ENCs (Figure 1A). More specifically, teachers projected that they would utilize the ENCs in planning future lessons (*M* = 89.92%, *SD* = 6.6%) significantly more than their prior application (*M* = 50.56%, *SD* = 19.6%). When examining teacher responses by combining responses across all three time points, concept 10 had the most utility (*M* = 82.9%, *SD* = 11.6%) and concept 2 the least (*M* = 60.5%, *SD* = 17.8%).

Given the significant interaction, the effect of time on rating scores for each ENC was assessed (Figure 1B). Statistically significant increases in the perceived relevance between the retrospective prior and future estimates of use were found for all ENCs except 5 and 10. The estimates of prior use were consistent with the pre-intervention data previously reported (Schwartz et al., 2019). For ENCs 5 and 10, their greater relevance to teachers at prior and current times prevented significant increases due to ceiling effects. Significant increases were also observed between retrospective prior and current application for ENC 2, suggesting this idea was initially the least familiar to teachers. We also observed significant increases between the current application and future estimates of applicability of ENCs 3 and 8. Average current application of each concept exceeded 50%, indicating teachers generally found relevance in all concepts for lesson planning. In summary, although there was a general tendency for the use of all 10 ENCs to influence lesson planning to increase over time, the acceptance and application rate varied with each ENC.

### Cross Case Qualitative Analysis

In planning the observed lessons and in the post-observation interviews, teachers discussed employing different ENCs. Teachers invoked 4 or 5 ENCs each, covering all ENCs. Among such a small sample, the frequency of ENC use cannot be generalized. Examples of how teachers applied each ENC, what was observed and how the teacher explained the connections appear in Table 2. The cross-case analysis looked beyond how individual ENCs were used (Table 2) and focused on identifying themes in the teacher observations and interviews that encompassed actions or thoughts across all ENCs. The overarching themes that emerged were the ideas that the ENCs influenced teachers’ thinking about their pedagogy and their views of students, and teachers’ planned and spontaneous actions in various classroom contexts (Table 3). The following discussion of examples supporting these themes includes the common idea that introduction of neuroscience ideas changed teachers’ views or practice.

**TABLE 2:** Examples of how teachers applied the educational neuroscience concepts.

ENC	Observed	Explanation	Change statement
1. Learning strengthens synapses. Remembering reactivates plasticity.	Ms. Able used a KWL chart to help students remember insect body parts.	I had never used the KWL chart before, but I used it specifically to help students to remember, so we went over it in the morning and when they left. I think my students used that chart and now they improved to a higher level than I expected.	I feel more confident. Before the class I knew [ENC 1] was important and after the class I wanted to make a greater effort to incorporate it into my lesson plan.
2. Different behaviors use different but overlapping circuits.	Ms. Bell gave students opportunities to wonder and investigate how to match graphs with correct equations.	Giving students the opportunity to look at the graphs, cut them out and key them into the graphing calculator and then place them with the correct equation was intentional to give them different experiences with the same concept.	The biggest difference is the confidence I have in it [ENC 2] being the correct approach. I am more intentional about using it.
3. Experiences and genetics shape circuit development.	The class used the Padlet app to compare graphs of different equations. Following Ms. Bell’s instructions, students shared what they noticed, what questions they had, and what they thought about asymptotes.	I am surprised at how much progress can be made by those I least expected it from. It [ENC 3] has taught me not to judge who will respond and who won’t.	
4. Rehearsal, application and self-evaluation lead to automaticity and mastery.	Ms. Able instructed students to use the information from the KWL chart to draw an insect and label parts. She asked students to talk about their drawings and the labels they wrote. When needed, she helped students sing the song to remember the body parts and transfer them to writing. She encouraged students to think about what they learned instead of just telling them answers.	I try to get students to apply what we’re learning in different ways. … I use self-evaluation now, especially asking them what they think about what they’re doing. I’ll ask a 3 year-old to evaluate their work.	I used [ENC 4] less often before the class, and now I use it more often.
5. Salience and repetition strengthen synaptic and circuit development.	Ms. Lake provided a graphic organizer, “Somebody Wanted/But/So/Then,” to help students remember the events of a narrative. She helped them read a story and then demonstrated on the board how to fill out the graphic organizer.	The plan was to repeat the lesson on [the] main idea and give them a visual [the organizer] that goes with it.	Now that I teach language arts, I realize the more important it is to use repetition - going back and evaluating what is going on and rereading the words, that sort of thing.
6. Emotions facilitate memory and decision-making.	Ms. Gomez gave students the opportunity to explore the concepts of equal and unequal by sharing a graham cracker with tablemates. The groups contained 1, 3, or 4 students. The groups were told to think of ways to share the cracker equally and draw their solutions. After breaking the cracker according to plans and observing the results in other groups, the students realized their pieces were not equally sized. After a discussion, students were given another chance. Results were better, but still some inequalities occurred because the sizes of the groups were different. Some students were frustrated or disappointed.	But I learned more about it [ENC 6]; made it [a] more salient idea and gave me more of an incentive to use it on a daily basis. We are so used to a toned-downed delivery. The lessons aren’t always experiential. We are concerned about how we engage them [students]. Usually it requires an interesting activity or experience that can be carried on over a period of days.	I knew of it [ENC 6], but I learned more about it – to use it on a daily basis.
7. Brain pathways, while similar across individuals, are shaped by unique experience.	Ms. Crow reminded students that they had previously learned to use a Venn Diagram to compare and contrast using hula hoops. In this class, she reviewed ‘compare and contrast’ by drawing a Venn Diagram on the board and using hair color. Students were divided into 3 groups and each group read a different version of Cinderella. Using a jig-saw structure, students then were placed in mixed groups where each student read a different version. They were given large pieces of paper to draw the 3 circle Venn Diagram and compare/contrast the 3 versions. Groups posted their papers on the wall for a gallery walk and class evaluation.	I think about how students learn with their own experiences and that they are unique … and learn by doing…. I realize students need to start small and use sensory before they move into the representational level. Now we start at the bottom and build up.	I did it [experiential learning] differently before taking the class, but now I know more after taking the class. … It makes sense to me to start at the beginning, sensory-motor…. Earlier I never thought how important it was to begin at the lowest level.
8. Physiology influences learning, memory and decision making.	One student did not want to participate in the jigsaw reading group. Ms. Crow gently reminded this student to share ideas and participate in the group discussion.	The student who did not want to participate has emotional issues. To yell won’t help, so I have to talk with him and be patient and calm. I give him alternative strategies to work on behavior. I try to be a role model for my students.	I knew [ENC 8] was an issue, but after [the class] I knew why. I have a lot more patience with my students because some are homeless or lack stable home lives.
9. Nervous system complexity produces reasoning, communication, creativity, curiosity.	At the beginning of the lesson, Ms. Gomez provided photos of whole items divided into parts with lines and items that had been physically separated into equal parts to help students see fractional relationships in both situations.	It [ENC 9] is what I tried to use at the beginning of the lesson. The pictures of each different item, one entire item and the one that had been sliced into pieces to see if they could organize/categorize the pictures. I want to step away and not tell them everything that is happening. I want them to tell me.	I used it [ENC 9] more after the class. I tie this concept to pattern recognition. I want students to come up with what’s going on.
10. Safe learning environments provide opportunities for deeper learning.	One student was not engaged in the group activity. After a quiet reminder and no improvement, Ms. Crow asked the student to step into the hall away from the class to calmly encourage the student to add ideas to the group’s Venn Diagram. She stood in the doorway while talking to the student so she could continue observing the other groups.	Every day I strive to create a safe learning environment with the student who didn’t want to participate. He felt he couldn’t participate because he didn’t know what to do. It is challenging to create this safe classroom environment.	I’ve always known a safe learning environment impacted students and their learning, but I didn’t realize why until after the class.

*Statements teachers made with respect to each ENC, explaining their instructional choice and a change in their practices are also included. The explanation and change statement columns contain quotes from the interviews regarding the vignette in the observed column.*

**TABLE 3:** Cross-case comparison of how the teachers applied the ENCs.

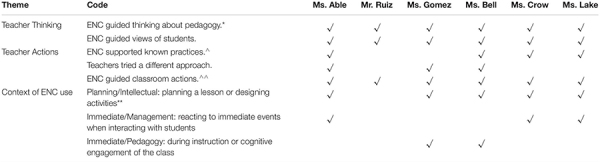

*This category includes evidence of the ENCs across a broad range of teachers’ thoughts; justifications, deeper understanding, guidance, motivation, confidence, beliefs, etc.

**This category focuses only on evidence for use of the ENCs in specific planning prior to the observed lesson.

*^∧^This category includes evidence of teachers’ reflections upon classroom practices they said they used previously. These practices may have been supported by an observation.*

*^∧^^∧^This category includes teacher narrative explanations which are consistent with the observations.*

#### Theme 1: Teacher Thinking

##### ENCs affected teacher thoughts about pedagogical decisions

Across all cases, ENCs guided teachers’ thinking about pedagogy. Teachers used the ENCs to reason why certain pedagogies were more effective, deepening their understanding of these approaches. For one teacher, this understanding illustrated how certain practices could be used. For another, the ENC affirmed or resolved prior concerns and doubts about specific practices.

Teachers thought about involving certain pedagogies in their lessons because ENCs helped them see why these pedagogies were effective. ENC 8 helped Ms. Able to acknowledge that students’ physiology when entering the classroom, whether through hunger, lack of sleep, stress or emotional state, influences their learning capacity. This understanding led to pedagogical decisions that respected students’ physiological and emotional needs. More specifically, in the observed lesson, Ms. Able welcomed each student upon their arrival and asked students to greet each other to “offset the problems they may bring from home.” She also allowed students to play with Legos to “keep them calm and stress-free” while waiting for everyone to arrive. She added, “I knew it [taking care of students’ emotional needs] was best for kids, but now I have a deeper understanding of why.” Ms. Crow indicated that the course has a similar impact on her understanding of ENC 8, “I knew before it [ENC 8] was an issue, but after [the course] I knew why… I understand that those [nutrition, hormones, stress, and sleep] are factors within the child’s learning.” Considering the influence of physiological and emotional status on students’ performance, Ms. Crow decided to be more patient in her interactions with them. In the observed lesson, a student was uncooperative. Ms. Crow’s explanation reflected her consideration of the student’s emotional needs, “To yell won’t help, so I have to talk to him and be patient and calm. I give him alternative strategies to help him with behavior.”

Educational neuroscience concepts also influenced teachers’ thinking about how pedagogy should be used. In discussing ENC 1, Mr. Ruiz said, “The neuroscience class did influence my use of repetition. I am more aware how repetition should take place.” He recognized that the student actions of repeating and remembering strengthened synapses as they learn. Mr. Ruiz told us that he used to focus repetition on mathematical algorithms but found that his students did not understand why they worked. In response, he repeatedly drew diagrams for students to copy, remember, and apply to later problems. He stated, “students are getting used to seeing me doing the exercises, the graphs and illustrations attached to descriptions of key words. They see a connection between what I say and how things actually work.” In addition, during his math class, Mr. Ruiz instructed students to take notes, draw diagrams, and make personal observations in their personal notebooks. These actions gave students opportunities to repeat the exercise themselves in diverse ways, activating plasticity.

Educational neuroscience concepts also affirmed teachers’ concerns about pedagogy. Regarding student engagement, Ms. Gomez pointed out, “We are so used to a toned-down delivery. The lessons aren’t always experiential, and we are concerned with how we engage them [students]. Usually it requires an interesting activity or experience.” She invoked ENC 6, justifying actively involving students in the observed lesson on fractions, “[The class] made it [ENC 6] more salient and gave me more of an incentive to use it on a daily basis… It is a lot harder to think about how you can create that [emotional] stamp… but I use at least one experience per unit or topic.” Ms. Gomez introduced the concepts of fraction using pictures of pizzas. She applied her deeper understanding to incentivize students with the opportunity to explore fractional parts using graham crackers and chocolate bars. Students realized the size of the portion was impacted by the number of students sharing the cracker. Students with the smaller portions were distraught; the outcome was unfair. These experiential activities provided students with rich emotional feelings that reinforced their memory of the lesson.

##### ENCs guided teachers’ views of students

As the ENCs described and explained the fundamental neuroscientific processes in learners’ brains, they enriched teachers’ general understanding of students. This broader view embraced students’ variability, unique backgrounds and capacities, and respected students’ physiological and emotional needs. Thus, guided by the ENCs, teachers more intentionally incorporated these insights into their pedagogical thinking as they crafted students’ learning experiences.

Educational neuroscience concept 7 helped Ms. Crow to appreciate that although learning the same content or skills would develop brain circuits for each student, the process may occur in unique ways. This idea further evolved into a new insight regarding different student learning progressions: “Students are unique. Some students don’t need sensorimotor [activities], some need representational levels, and others need to work on the abstract level.” Such a view of the students’ learning process encouraged her to include activities that prepared students with more concrete experiences before transitioning to more abstract content. In her math lesson, Ms. Crow used actual hula hoops to help students understand the more abstract concept of Venn Diagrams, “I realize students need to start small and use sensory [activities] before they move to the representational level. Now we start at the bottom and build up.”

Educational neuroscience concept 8 affected Ms. Lake’s view of how physiological and emotional needs impact learning, “…[I] always felt the emotional status and the state the students are in make a huge impact on what they are doing… There is a bigger impact than we realize…nutrition, hormones, stress; all these factors affect the students.” This view of students also led to a pedagogical decision in the observed lesson. At the beginning of class, a student was compelled to tell about a lightning strike in his neighborhood. Ms. Lake recognized his need to process and share that emotional event with his classmates, “If they are upset, they are not going to listen.” Guided by this understanding, she let him finish the storytelling before starting with the lesson. Moreover, her awareness of the impact of safe learning environments extended beyond her classroom: “I knew that safe learning was important, but now I understand that it is essential to know when kids are being picked on in the halls. The effect on deep learning surprises me.”

In addition, teachers noted their ‘surprise’ at the abilities of their students, after applying ENCs to practice. Teachers often hold an expectation of what their students are capable of, given their age, grade, and previous performance. Implementing pedagogies aligned with ENCs provided teachers an opportunity to see the potential of their students in a new light.

In discussing ENC 4, Ms. Able acknowledged that for her students to master what they were learning, they needed to constantly reinforce their synapses by approaching the material multiple ways, through rehearsal, application, and reflection. Thus, in her pedagogical plan of the lesson on insects, she aimed “to get students to apply what we’re learning in different ways.” She asked students to apply their knowledge of insects by singing, drawing and writing. She also asked them to discuss what they learned about the insects and reflect upon it. As a result of the practice, her view of her pre-K students changed, “It [pedagogy aligned with ENC 4] pushes them to higher level thinking. They are talking more and using vocabulary more often…. You ask a three-year-old to self-evaluate, and you don’t think you’ll get a good response, but they actually like it…they know if they have done their best. It is surprising in a good way.”

The same occurred to Ms. Gomez, who obtained the idea from ENC 9 that learning should involve complex cognitive processes such as reasoning and communication. She associated this idea with a pedagogy with which she “want[s] students to come up with what’s going on.” and “verbalize what they think.” Multiple times during the observation lesson, Ms. Gomez required students to turn to a partner to share their thinking. Then the students were asked to share with the class what they noticed from the activities in the lesson. Students’ performance reshaped her idea of their ability, “Every time I use it [ENC 9], they surprise me with their answers - their ability to notice certain things. I think they won’t notice this, but they do.”

In a similar event, Ms. Bell noticed math anxiety in her students, and said, “Students come into this class believing they don’t like math or can’t do math. They don’t have confidence and say, ‘I’m not a math person.”’ Guided by ENC 3, Ms. Bell embraced the idea that experience plays a pivotal role in learning. This idea strengthened her confidence in making her pedagogical decisions when addressing the math anxiety in her students, “There’s an underlying idea that students need to be given complicated work, [but] I need to make the learning accessible so that they can have the experience that tells them they can do this.” As a result of her effort, students in Ms. Bell’s class impressed her with their potential, “I am surprised at how much progress can be made by those I least expect it from. It [The students’ progress] has taught me not to judge who will respond and who won’t.”

#### Theme 2: Teacher Action. ENC Supported Known Practices, Encouraged Untried Approaches and Generally Guided Pedagogy

Beyond influencing teachers’ views of students and thinking about pedagogy, the ENCs also influenced teachers’ classroom actions. Intellectually, teachers connected the ENCs to pedagogical practices such as repetition [ENCs 1, 4 and 5], safe learning environments [ENCs 8 and 10], addressing students’ emotional needs [ENCs 6, 8], providing students with agency [ENCs 1, 3–7, 9], and associating new learning to existing knowledge [ENCs 1–5, 7, 9]. They had learned about, knew of, and to some extent used most of these practices before taking the course. The course did not instruct teachers in the use of a set of “novel” practices. Instead, teachers made connections between the ENCs and the pedagogical practices modeled during the course. During classroom observations, the influence of the ENCs on teachers’ pedagogical decisions were evident as teachers reflected upon their actions in the subsequent interviews. Teachers were observed to enact previously known pedagogies, and to try new or untried approaches. The ENCs generally guided sequences of teacher actions.

In a more pragmatic fashion, teachers invoked various ENCs in their use of pedagogies that were already familiar. Concerning ENC 4, Ms. Able indicated that having students apply their prior knowledge in learning new lessons and evaluate their progress was part of her practice, “I used it less often before the class and now I use it more often.” Ms. Crow, related ENC 7 to matching activities to students at different stages of development, “I have done that before, like I’ve done differentiation where students discover their own learning.” In similar fashion, Ms. Bell applied ENC 2, expressed that she had previously thought about integrating multiple different activities for students to learn about functions in her lesson, but had concerns about the investment of time for her set of activities. “The biggest difference is the confidence I have in it [now] being the correct approach.” As for ENC 5, Ms. Lake told us that having students repeat what they have learned to improve memory has been a conventional practice for her, “I’ve worked with Special Ed for 15 years so it is not surprising. It is more science to go with the things that I have done. Like affirmation.”

In other instances, teachers adopted previously untried approaches. Their understandings of why these pedagogies might be effective had been bolstered by the ENCs. Teachers were now confident that they could successfully utilize the novel pedagogies. For example, Ms. Able indicated that ENC 1 had motivated her to focus on the importance of helping students remember what they had learned, so she tried using a KWL chart. Ms. Able said, “I had never used the KWL chart before. I used it [chart] specifically to help students remember. My students used that chart, and they have improved to a higher level than I expected.” Relating to ENC 9, Ms. Gomez knew that she wanted her students to express their own ideas because, in her own words, “the learning emerges from them.” She added, “but it wasn’t until I saw CRP [Critical Response Protocol – a strategy modeled in the course (Ellingson et al., 2016)]; that technique allowed me to see how I might use it [ENC 9] with my students.” After the observed class, Ms. Gomez expressed her passion in utilizing this new approach, “I don’t use it [CRP] just in math but also in reading and writing.” Ms. Bell wanted her high school students to investigate the relationships between functions and graphs [ENC 2] so she devised an activity to physically sort and match the equations to the graphs. “The previous year, we gave them the equations and the graphs already graphed side by side. So they didn’t get the chance to see all the graphs and wonder about them and investigate, ‘what did they notice.’ It was a more static and teacher-directed lesson.”

More generally across all the interviews, teachers acknowledged at least one instance in which they applied an ENC as a principle to guide some aspect of their observed classroom actions. Ms. Lake recognized the importance of using repetition (ENC 4) when she kept reviewing the story line as she guided students to analyze and evaluate characters’ motivations. Acknowledging the importance of having the students remember math strategies and procedures, Mr. Ruiz guided them in their note taking (ENC 5). Comparing the other teachers’ observed actions and subsequent discussions revealed that different ENCs can lead to similar pedagogical choices and a single ENC can be applied in a variety of ways.

Ms. Bell and Ms. Gomez both chose to incorporate a sequence of experiential, student-centered activities in their lessons, but they attributed their pedagogical decisions to different ENCs. Ms. Bell gave high school students a more active role in exploring and interpreting the graphical shapes of rational functions through matching graphs with the respective equations and function tables and confirming their choices using a graphing calculator. Students then used a sticky note web app to share their observations, pose questions, and consider alternative points of view. She explained the influence of ENC 1 by saying, “I’m mindful that every new experience, idea, thought, changes the brain… so by making them experience the lesson, it opens up that plasticity.” Ms. Gomez used ENC 6 to guide the actions she took to engage first graders and provide an emotional impact. During the observed lesson on equality and fractions, Ms. Gomez challenged the students to divide a single graham cracker equally among their three or four tablemates. After agreeing on a plan and then dividing the crackers, students rotated around the tables and realized that tables with three students had larger cracker pieces than those with four students. By the end of the lesson, students could explain the concept of equal and unequal using the varied sizes of cracker pieces in a more personal way than if Ms. Gomez had used paper cut-outs of fractional shapes. She stated, “The lessons aren’t always experiential, and we are concerned with how we engage them [students]. It [engagement] requires an interesting activity or experience… it is a lot harder to create that stamp.” Both Ms. Bell and Ms. Gomez invited students to share their thoughts and reflect on their learning with partners and the class. A common series of actions was seen in both teachers’ classroom practice: reviewing prior knowledge (functions or concepts of equal and unequal), providing students with equipment (graphs or crackers), encouraging students to apply their prior knowledge in problem-solving (graphing a new function or splitting crackers equally), engaging students in various learning behaviors, inviting students to share their thoughts with partners or the class and encouraging students to evaluate and reflect on their learning. Although the activities are unique, both their actions focus on students in offering them a learning experience that “changes the brain (Ms. Bell)” and “creates that [emotional] stamp (Ms. Gomez).”

Both Ms. Able and Ms. Crow created environments where students felt safe physically and emotionally (ENC 10). Mrs. Able created a safe learning environment by going over the classroom rules daily, reminding the pre-K students to walk in the classroom, and leading students to think about why walking was a necessary rule. By understanding the importance of predictability in a young child’s life, Ms. Able commented, “The schedule is consistent, and the rules are clear.” In contrast, Ms. Crow organized her actions to respond to an individual fourth-grade student who was disengaged from the group he had been assigned to join. Initially, Ms. Crow whispered an encouragement to participate in the group activity. When the student became disruptive, instead of reprimanding him in front of his peers, the teacher quietly asked the student to step out into the hallway to better understand the reason for his reluctance to participate. In the privacy of the hall, the student shared that he did not know how to contribute to the group’s assignment. She offered suggestions, and the student was able to return to the group without further issues. When asked about ENC 10, Ms. Crow indicated that until taking the course she did not understand how a safe learning environment impacted students and their learning. Now she considers it on a daily basis. In both cases, Ms. Able and Ms. Crow organized their actions according to their unique situations and ensured that students felt safe in their classroom physically and psychologically.

#### Theme 3: Context. Teachers Used ENCs to Respond in Different Contexts

The ENCs influenced teachers’ practice across various educational contexts. For some teachers, the ENCs played a role when they were planning the lesson or designing activities. For others, teachers invoked ENCs in their decisions reacting to immediate events when interacting with students or guided teachers’ immediate pedagogical decisions that deviated from the original lesson plan. Moreover, the ENCs guided the way teachers gave instructions or cognitively engaged the class. Immediate and contextual ENC uses were not as prominent as teacher thinking and actions.

Ms. Gomez and Ms. Able made it explicit in the interview that ENCs were influential when they were trying to decide on a pedagogy or activity. Ms. Gomez indicated that she invoked ENC 1 in her thinking, justifying why reviewing what students have previously learned was included in the lesson plan. “Depending on the subject… I try to attach the idea of prior knowledge in every lesson.” When explaining her plans for the math lesson, she said, “We had learned about equality and inequality at the beginning of the year. One side has to equal the other side. Having them [students] remember that definition would help the synapses get stronger.” Similarly, Ms. Able discussed using ENC 5 in planning to practice vocabulary, “I tried to plan for opportunities for them to repeat the vocabulary words like singing that song many times. That was one way to get them to repeat and practice… I definitely plan for those opportunities more often.”

Sometimes the lessons did not unfold as teachers planned, when students were not engaged in the activities. Teachers need to react to these immediate classroom events, to manage the classroom appropriately or to shift gears and revise the lesson in real time to adjust to student needs. As described earlier, Ms. Able, Ms. Crow and Ms. Lake offered examples in which ENCs affected their immediate reactions to disruptive behavior. Ms. Able acknowledged the importance of maintaining a safe learning environment (ENC 10), preventing unwanted injury or chaos from running. Likewise, Ms. Crow was sensitive to a disruptive student’s emotional needs (ENC 8), and provided him with a path forward. Ms. Lake also appreciated the emotional needs of students (ENC 8), and decided to let a student share with the class a lightning tale before transitioning to the lesson.

A third educational context in which teachers used ENCs to guide their thinking and actions was when they were trying to cognitively engage the class during instruction. This context was different from lesson planning because it did not happen before the class, but during the class. In lesson plans, teachers speculate on how their student would respond to the activity without knowing how they actually react. During the instruction, teachers need to quickly respond to students’ reactions and help them to cognitively engage in the learning. In the during-instruction contexts, teachers need to be observant of subtle problems that may undermine students’ learning experience and process. The ENCs also offered important insights into how teachers adjusted their pedagogy in this context. For example, over the course of the lesson matching functions to graphs, Ms. Bell wanted to have students share their thoughts to see what others observed using a sticky note app. Once she observed the way her students performed in that activity, Ms. Bell reoriented students’ thinking to a deeper level by intentionally modifying the instruction, “That was another thing I changed, from ‘I wonder if’ to ‘I noticed’… so students reflect on what other students saw and think about what they thought.” This change was guided by ENC 4 that highlights the importance of self-evaluation to learning.

Overall, the ENCs affected the way that teachers selected pedagogy, organized lessons when planning the lesson, reacted to unpredicted immediate classroom events, and managed to maintain students’ cognitive engagement in the class activity during instruction. We did not observe instances where the ENCs guided teachers responding to student misconceptions or terrific insights by spontaneously changing their pedagogy leading to a fruitful tangent.

Across all themes, teachers viewed the neuroscience ideas as applicable to their classroom decision making and practices in broad general terms. These ideas provided approaches, justifications, affirmations or resolutions to problems that arose in their classrooms. In the words of Ms. Gomez,

*“It’s [the ENCs are] all information we can use. It is hard for me to pick out one thing…. It all influences my teaching as a whole as it affects delivery…. It gives me a mental checklist to go through as I plan. One of the strongest impacts that learning about these nervous systems concepts have made are in accepting or rejecting certain teaching methods, practices, lesson delivery, … frameworks. Sometimes we are given mandates in how to deliver instruction, but now I find I have better ways to teach, and sometimes I ignore mandates that I know won’t work. I say research shows that those activities aren’t effective.”*

### Disconfirming Evidence

Across the six classroom observations, we found varying instances of the application of ENCs. Half of the teachers applied the ENCs liberally throughout their lessons. One teacher applied the ENCs a moderate amount in her observed class. Two teachers had many fewer classroom instances which were influenced by the ENCs. This could be attributable to many unexplored reasons, from lesson goals and content to available time. All teachers endorsed some use of the ENCs as guides to their practices during the interviews. In the summative interview responses, 4 of the 6 teachers stated explicitly that the ENCs were important to their teaching practices. The two teachers who did not respond this way were also the two teachers who had fewer classroom examples that they linked to an ENC. Rather than arguing forcefully that the ENCs did not apply, the more neutral endorsement by these two teachers may represent early stages of application.

## Discussion

### Significant Findings

The survey and subsequent observations with interviews provided different perspectives on how the teachers viewed the ENCs. The surveys revealed that a year after PD, non-science teachers unanimously found the ENCs useful as organizing principles in their pedagogy. The observation-interview process demonstrated that the ENCs influenced teachers’ views of students and informed their classroom pedagogy and actions. Collectively the ENCs may have contributed to what might constitute a neuroscience framework for approaching pedagogical decisions that allowed teachers to plan, act, think and respond in dynamic ways in and out of their classrooms.

The surveys demonstrated that teachers found some ENCs more useful than others. However, all were deemed relevant. All played an active role in the teachers’ thinking about lesson design. Teachers indicated they were currently applying the ENCs and intended to continue to use them in the future as guiding principles. As the results represent the experiences of teachers of different ages, grade levels and subjects, the ENC’s relevance appears to be stable over a year and useful in a variety of educational contexts.

Classroom observations and interviews offered a more nuanced view of how the ENCs impacted participant thinking and actions. Three themes summarized how the ENCs influenced teachers’ (1) thoughts about pedagogy and students; (2) actions in planning and execution of lessons; and (3) responses to events in and out of the classroom. These themes highlighted the principal ways in which the ENCs influenced teachers’ pedagogical decisions in real time as well as in lesson planning.

Like other theories provided by developmental psychologists that act as frameworks (Beloglovsky and Daly, 2015), the ENCs provided a rational basis for making pedagogical decisions. Thus we consider that they may act as a framework for exploring pedagogy. We did not examine whether the ideas provided in the ENCs replaced or competed with other more traditional educational theories. The ENCs should complement rather than supplant prior theories by providing the biological basis for educational psychological findings (Diamond and Amso, 2008). The ENCs were designed to summarize important neuroscience concepts that teachers should understand (Dubinsky et al., 2013). They were not designed to describe developmental progressions or behavioral interventions. Teacher responses in the current analysis support the idea that the ENCs may be useful as a set of guidelines, or framework, for making pedagogical decisions both in planning and real time.

### The Role of ENCs in Teacher Pedagogy

The three themes emerged through the teachers’ use of a number of different ENCs, providing them agency and insight (Table 3). The first theme, Teacher Thinking, was illustrated through two perspectives: the teachers’ current or updated thoughts about their pedagogy or their views of students. Noteworthy, this was the only theme where the ENCs influenced all six teachers through both perspectives. The second theme, Action, characterized how the ENCs influenced teachers’ classroom practices through supporting known pedagogies, encouraging them to try new pedagogies and generally guiding sequencing or pedagogical choices. Teachers took the ENCs into account as they prepared, organized, or sequenced activities prior to encountering students (Table 3). All six teachers identified ways that the ENCs supported or guided their classroom actions. Four of the six teachers specifically pointed out how the ENCs supported practices they considered using or were encouraged to continue using. Half of the teachers claimed they tried a different approach because the ENCs justified the change. Again, all teachers used one or more ENCs as the basis for a specific action or change they made. The third theme, Context of ENC Use, reflected how ENCs were applied in lesson planning or how they helped teachers respond to emerging classroom issues. Multiple examples across five of the six teachers highlighted situations where their thoughts about pedagogy were directly supported by observed responses to specific management issues or the need to make instructional changes while engaging with students. The flexibility and power of the neuroscience framework emerged from the unique ways that teachers applied the ENCs in real time to behavioral or pedagogical challenges (Table 3).

The teachers used the ENCs to guide their classroom actions as well as respond to student needs. The observations and interviews revealed how teachers invoked various ENCs to justify new pedagogical approaches, classroom goals and methods. Teachers demonstrated insight into the nature of student problems and how to increase student agency. They used the ENCs to justify changes in lesson plans and strategically choose pedagogies, as Ms. Gomez noted, “Sometimes we are given mandates in how to deliver instruction, but now I find I have better ways to teach.” The ENCs contributed to a framework that helped teachers explain student behaviors and understand the impact of students’ emotions on learning and development. While the ENCs were not prescriptive in terms of dictating specific actions, they helped teachers organize actions in response to specific contexts.

Additionally, we cannot claim that this set of ENCs is the most parsimonious in generating similar results. The current set of ENCs were honed through multiple experiences to provide a foundation for teacher PD (Dubinsky et al., 2013, 2019). Neuroscience topics and activities that did not deepen the understanding of learning and memory (for example sensory transduction) were removed from iterations of similar neuroscience teacher PD (MacNabb et al., 2006; Roehrig et al., 2012). Some ENCs resonated more with teachers than others. Unpacking the relevance of any particular ENC to the overall framework may be possible in future research. A more nuanced view proposes teachers are not responding to the framework as a whole, but rather to individual ENCs that resonate with them. However, across this diverse group of non-science teachers, we observed no one-to-one correspondence between ENCs and specific pedagogies. Rather, the teachers used the ENCs to guide a broad range of classroom actions.

Collectively the ENCs represented a body of foundational understanding about the brain that increased teacher agency. Teachers made their own connections or translations between the ENCs and their own practices. They shifted their focus from the lesson, classroom management and organization to the students’ needs, issues and success. Teachers recognized the importance of the students’ experiences and desire to be agents of their own learning. This shift was revealed when the teachers discussed and synthesized how neuroscience might impact their practices rather than having the ENCs prescribe specific classroom actions. Evident among all teachers was the neurological basis underpinning their understanding of student behaviors, needs, emotions and states of mind. Comparably, elementary teachers who participated in a 2-year lesson study program framed by neuroscience theories deepened their understanding of student knowledge construction and could justify their pedagogical decisions through a neuroscience lens (Tan and Amiel, 2019; Tan et al., 2019). After a course in neuroscience, Israeli teachers similarly embraced neuroscientific justifications for pedagogical choices and increased their support of individual students’ needs (Friedman et al., 2019). In their final comments, the interviewed teachers emphasized the influence of the ENCs as a frame for viewing student learning, growth and progress, and how to integrate views of students with effective pedagogy. As Ms. Bell noted, lesson plans not only need to address, “…all the elements in a lesson plan” but “…what is important from the standpoint of the students.” Viewed more systematically, Ms. Crow emphasized that “policy makers need to know about the brain and how students learn. … Sometimes teachers are not flexible and teach the same way they’ve always taught.” Thus, foundational neuroscience knowledge acted as a framework to help teachers develop both their pedagogy and views of students.

### Rival Hypotheses

Unlike experimental designs, the case study cannot rule out all alternate explanations, yet plausible competing hypotheses can still be addressed to increase the certainty of conclusions (Yin, 2011). Study limitations are also considered along with the alternative hypotheses. The prerequisites of having attended the course and consenting to be observed limited the sample size. Since the teachers taught in diverse settings, the six cases compared here are in line with the recommendation for 6–10 cases for a purposive cross-case analysis (Malterud et al., 2016). The small sample size of this case study and its lack of controls prevented generalization of the findings. However, we can rule out the null hypothesis that the ENCs, as a framework, had no lasting impact on teachers’ pedagogy. The survey results clearly demonstrated the relevance and importance of the ENCs to teachers a year later, which are confirmed in interviews. All teachers highlighted the impact of the ENCs in their thinking, actions, and sensitivity to their students’ learning needs. Teachers embraced and internalized a deep understanding of how learning takes place. As one teacher expressed, “I really owned it [synaptic plasticity] after taking the Neuroscience of Educators Class.”

Threats to validity of the study include selection bias, context and interactions between the teachers and researchers. The teachers self-selected in choosing to attend the Neuroscience for Educators course and the MBE program. Thus, they were predisposed to want to learn neuroscience. While we observed that this diverse group of non-science teachers all used the ENCs to guide their classroom actions, whether the ENCs can act as a framework for all teachers is unclear. Furthermore, context matters (Fischer and Bidell, 2006). Teachers who are struggling, facing shortages of resources or absence of support may view the ENCs differently than teachers who are currently satisfied with their instruction methods, have all the resources and support they want and generally enjoy their students and their jobs. To understand the impact of the ENCs in teacher training better, we recommend that future research focus on larger samples, in diverse contexts with teachers who are not self-selected, as in this study. Although there is a consistent trend that neuroscience knowledge positively influences teacher pedagogy across different countries with both pre- and in- service teachers (Tan and Amiel, 2019; Tan et al., 2019; Howard-Jones et al., 2020; Luzzatto and Rusu, 2020; Brick et al.,2021a,b), we can’t comment on how well the ENCs serve teachers who are currently satisfied with their approach to teaching and their work environment. Lastly, interactions between the teachers and the researchers who were embedded in the research as instructors could have influenced the outcomes. Teachers might want to please instructors. However, the teachers were generally very open and honest about where and when experiences did or did not resonate with their thinking and practice. The amount of ENC application varied among the teachers (Table 3). In regard to ENC 3, Ms. Bell said, “Honestly I’m not following the meaning of #3.” If an ENC felt redundant to previous training, teachers also told us. Referring to ENC 5, Ms. Lake did not inflate the importance of the ENCs saying, “I’ve worked with Special Ed for 15 years so it [ENC 5] is not surprising… It is more science to go with the things [teaching strategies] I have done… like affirmation.”

One competing hypothesis may be that the ENC framework only confirms what educational researchers have already demonstrated as best teaching practices. But that conclusion may be too categorical given that all the teachers interviewed claimed that their views of students had changed as a result of the neuroscience training. Furthermore, the teachers discussed their interest in understanding the reasoning behind the need to implement best practices. The ENCs provided explanations that teachers used to justify implementation of certain strategies or choice of one strategy over another. Owens and Tanner (2017) provide a detailed description of how neuroscience can explain the efficacy of the think-pair-share strategy. Similarly, the ENCs provided the current teachers with a neurobiological explanation for why best practices work.

A second alternative hypothesis would be that the active learning incorporated into the PD experience produced the changes in teachers’ practices, rather than the neuroscience content. In this case, teacher justifications should have been that they liked what they experienced and were trying to imitate that. The pedagogy used in the PD was consistent with best PD practices (Garet et al., 2001; Desimone, 2011; Darling-Hammond et al., 2017). We further argue that the nature of the pedagogy used to present the ENCs should be consistent with the ENCs. Resolving this issue would require further controls examining what specific influence the pedagogy used in the PD has on classroom implementations, as opposed to the neuroscience content.

The third alternative hypothesis is that previous training may be playing decisive roles in the observed changes. These teachers were currently or had previously enrolled in other courses in their master’s program or may have experienced other PD elsewhere. Such experiences were expected to be diverse but could have contributed to their individual process of change. Several teachers did mention how ideas from their master’s program resonated with this course. Such overlapping experiences are consistent with the possibility that teachers are continuing to integrate their understanding of how students learn and that the outcomes observed here are related to the program more than to a single course. Only a stand-alone PD experience could rule out any overlap; yet in the great majority of cases, teachers consistently used neuroscience to justify, explain or apply a practice with what they claimed was deeper understanding, more motivation or greater confidence. In a similar fashion, only a control group could allow us to rule out if the natural growth of teachers responding daily to the needs of their students would have brought them to the same conclusions they reached after this course. Thus, the boundaries are fuzzy that distinguish where experiences are unique and account for the reported observations vs. when they are complementary and resonate, leading to further growth.

### Current Thinking and Discourse

Feiler and Stabio (2018) characterized the relationship between neuroscience and education over the last three decades as proceeding along three themes: application, collaboration or translation. Where the effort focused on the application of neuroscience to education, the goal was to find ways to directly inform practice based on neuroscience findings. However, the responsibility for this effort has created issues of agency. When researchers assume the responsibility, we characterize the effort as “researcher-initiated.” Han et al. (2019) model this approach by assembling interdisciplinary research teams representing multiple perspectives to find where and how neuroscience can inform educators. In contrast, when the responsibility shifts to teachers for finding applications, we characterize this effort as “teacher-initiated,” which increases their agency. In general, we expect that application research will continue to offer insights on how the brain supports academic behavior in areas such as math, reading or executive control (Bunge et al., 2002; Gabrieli, 2009; Lyons and Beilock, 2012; McNorgan, 2021); but whether researchers or teachers are responsible for finding ways to apply insights to education will impact teacher agency. Alternatively, collaborative relationships (Feiler and Stabio, 2018) seek a more even contribution from educators and researchers where all parties collaboratively define and address challenges of interest. This arrangement assumes that the outcomes are greater than that from any individual contribution. This approach has inspired the creation of research school networks or models of collaboration similar to hospitals preparing interns with the goal of providing teachers the necessary time, experience and practice to build personal meaning out of complex ideas so that they can use them responsibly and meaningfully (Fischer, 2009; Schwartz and Gerlach, 2011). Ultimately graduates of such programs are highly skilled in research methodologies, the epistemologies of different disciplines as well as the content each discipline generates. While some graduates return to the classroom to leverage these skills, others are recognized as experts in their school districts or their communities. They are emerging as a new class of professionals, neuro-educators, skilled at scaffolding or mediating conversations to define the value, purpose or potential in new neuroscience research (Schwartz and Gerlach, 2011). In this regard, these neuro-educators can act as agents in supporting Feiler and Stabio (2018)’s last theme, translation, where the goal is to make neuroscience research more accessible to educators to improve teaching and learning. However, this theme transfers the responsibility of understanding and applying neuroscience concepts to the classroom from researchers to a new class of experts, which has the same effect of undermining teacher agency.

Fitting between these tiers is PD that focuses on increasing teacher agency by providing relevant neuroscience knowledge to education, as explored here. While the time commitment is shorter than the collaborative efforts described earlier by Feiler and Stabio (2018), this PD must still ensure that the neuroscience is accurate and not misconstrued, and provides teachers the time to develop a personal understanding of relevant neuroscience concepts so they can identify their value in their own contexts (Dubinsky et al., 2013; Tan and Amiel, 2019; Tan et al., 2019). To explore the feasibility of the teacher-initiated approach, we provided teachers with neuroscience knowledge and the opportunity to discuss its connection and application to their practices. Then we followed-up with them after a year of teaching to explore their thinking about the relevance of a set of foundational neuroscience concepts (the ENCs) to their practices. The teachers applied the neuroscience ideas in diverse ways to their planning and classroom implementation of lessons, reinforcing known or encouraging untried pedagogies in a variety of contexts. While neuroscience did not dictate specific practices, it provided teachers with a knowledge basis for making pedagogical choices, in advance or on the spot in class. In this way, the ENCs may have acted as a framework for evaluating and understanding what constituted best classroom methodologies.

Shulman outlined seven different kinds of knowledge that teachers needed in their profession: content knowledge, general pedagogical knowledge, pedagogical content knowledge, theoretical knowledge of educational philosophies/theories, knowledge of the curriculum, knowledge of educational systems, and knowledge of students (Shulman, 1987). Researchers who otherwise argue appropriately for which neuroscience concepts are relevant to education may overgeneralize when they assert that neuroscience directly impacts pedagogical knowledge (Ansari et al., 2017). They forget that educational research, not neuroscience research, determines best classroom practices. Neuroscience provides the foundational knowledge of what goes on in the brain as one learns. This falls clearly into Shulman’s category of knowledge of students, which included their physiology and development (Shulman, 1987). In the current study, teachers also conveyed that neuroscience changed how they viewed their students, indicating a growth in their knowledge of students. Teachers utilized this (neuroscience) knowledge of students to choose appropriate pedagogies, whether content specific or general, from their own pedagogical knowledge. Neuroscience may have provided a framework upon which the teachers could prioritize and make appropriate pedagogical decisions. Thus, neuroscience supplied teachers with usable knowledge that they could apply in their practices. These results satisfy Clement and Lovat (2012)’s criteria establishing the relevance of neuroscience to education.

## Data Availability Statement

The raw data supporting the conclusion of this article will be made available by the authors, without undue reservation.

## Ethics Statement

The studies involving human participants were reviewed and approved by the IRB of the University of Texas at Arlington. The patients/participants provided their written informed consent to participate in this study. Written informed consent was obtained from the individual(s) for the publication of any potentially identifiable images or data included in this article.

## Author Contributions

MS, JD, VH, and ZC contributed to conception and design of the study, performed the qualitative and cross-case analysis, interpreted the results, contributed to manuscript drafting and editing, read, and approved the submitted version. JD and VH conducted the course, collected the data, and transcribed interview and observation data. ZC performed the statistical analysis on the survey data. All authors contributed to the article and approved the submitted version.

## Conflict of Interest

The authors declare that the research was conducted in the absence of any commercial or financial relationships that could be construed as a potential conflict of interest.

## Publisher’s Note

All claims expressed in this article are solely those of the authors and do not necessarily represent those of their affiliated organizations, or those of the publisher, the editors and the reviewers. Any product that may be evaluated in this article, or claim that may be made by its manufacturer, is not guaranteed or endorsed by the publisher.
