# Supplementary material for: Neuroscience Concepts Changed Teachers’ Views of Pedagogy and Students
Source: Front Psychol. 2021 Aug 11;12:685856. doi: 10.3389/fpsyg.2021.685856 (PMC8384951; doi:10.3389/fpsyg.2021.685856)
Supplement: Supplementary file 1 [file Data_Sheet_1.docx]

**Neuroscience for Educators**

**Supplementary Table 1**: Educational Neuroconcepts used in this study (Dubinsky et al. 2013; Schwartz et al 2019).

1. Learning strengthens synapses. Remembering reactivates plasticity.
2. Different behaviors use different but overlapping circuits.
3. Experiences and genetics shape circuit development.
4. Rehearsal, application and self-evaluation lead to automaticity and mastery.
5. Salience and repetition strengthen synaptic and circuit development.
6. Emotions facilitate memory and decision-making.
7. Brain pathways, while similar across individuals, are shaped by unique experience.
8. Physiology influences learning, memory and decision making.
9. Nervous system complexity produces reasoning, communication, creativity, curiosity.
10. Safe learning environments provide opportunities for deeper learning.

**All-Teacher Survey**

Directions:

We are seeking your ongoing help in assessing the impact of your summer course *Neuroscience for Educators*. Our goal is to analyze the course’s impact, and to improve its focus and potential for you, future educators and researchers. To that end, we would like you to appraise the extent to which you “applied” each of the ten neuroconcepts we introduced during the summer session.

In this context, *applied* refers to influencing the design of the lesson, the way you interacted with students, the way you executed the lesson, your expectations of the lesson or students, etc.).

Use your current understanding of each of the 10 neuroconcepts below to answer each question.

* <# and text of ENC>

1. Prior to the course *Neuroscience for Educators*, in what % of your lessons did you apply this concept? (slider response, 1-100)

b. In the 2016-17 academic year, in what % of your lessons did you apply this concept? (slider response, 1-100)

c. How likely are you to continue applying this concept in future lesson planning? (slider response, 1-100)

d. If you are unlikely to apply this concept, what are the obstacles preventing its application? (open ended response)

Repeat from * for each ENC, going from ENC 1 to ENC 10.

**Neuroscience for Educators**

**Post-observation, Structured Interview Questions**

The following questions will be asked for each neuro-concept you indicated would be applied to the lesson.

*According to your pre-observation form, you applied Concept #X <text of the ENC>. Is this a pedagogical approach you used before taking the Neuroscience for Educators course?

If yes, I used it before taking the class:

Are you using it in the same way/frequency, or is there a difference in the way you used it before taking the course compared to how you use it now? If there is a difference, explain how you changed/improved the way you applied the neuro-concept.

If no, I did not use it before taking the class:

Did taking the Neuroscience for Educators class directly influence your decision to include it in today’s lesson design? If not, why did you choose to include it?

Continue with questions 1-5 for each ENC:

1. What impact did this neuroconcept have on your lesson?

2. Did the inclusion of the neuroconcept create new issues or problems for you? What and How?

3. How confident do you feel using the neuroconcept?

4. How much have you pursued this style of pedagogy? (how often and to what depth)

5. Is this neuro-concept playing out the way you expected? What did you expect to happen? What surprised you? What would you change?

Repeat from * for each ENC on the pre-observation form.

Final Question

6. Which neuro-concepts are most likely to influence your pedagogy now that you have had opportunities to experiment using them? Which ones are most difficult to apply?
